# Cinchona Alkaloid Derivative-Catalyzed Enantioselective Synthesis via a Mannich-Type Reaction and Antifungal Activity of β-Amino Esters Bearing Benzoheterocycle Moieties

**DOI:** 10.3390/molecules19043955

**Published:** 2014-04-01

**Authors:** Han Xiao, Fang Wu, Li Shi, Zhiwei Chen, Shihu Su, Chenghao Tang, Hongtao Wang, Zhining Li, Meichuan Li, Qingcai Shi

**Affiliations:** 1 Key Laboratory of Green Pesticide and Agricultural Bioengineering, Ministry of Education, Guizhou University, Guiyang 550025, China; E-Mails: fangwu90@163.com (F.W.); s2012l@sina.com (L.S.); yan.zhilie@163.com (Z.C.); sushihu999@gmail.com (S.S.); chtang1122@163.com (C.T.); wanghongtao1115@126.com (H.W.); aning072@126.com (Z.L.); lmcuvhn1987@163.com (M.L.); shiqingcai1988@163.com (Q.S.); 2 School of Chemistry and Environmental Science, Guizhou Minzu University, Guiyang 550025, China

**Keywords:** β-amino acid ester, antifungal activity, benzoheterocycle, enantioselective synthesis

## Abstract

An efficient synthesis of highly functionalized chiral β-amino ester derivatives containing benzothiophene and benzothiazole moieties is developed by a Mannich-type reaction using a cinchona alkaloid-derived thiourea catalyst. The desired products were obtained in good yields and high enantioselectivities (~86% yield, >99% ee) using to the optimized reaction conditions. The synthesized compounds were characterized by ^1^H-NMR, ^13^C-NMR, IR, and HREI-MS analyses. The bioassays identified that compound **5dr** has excellent antifungal activity, with a 60.53% inhibition rate against *F*. *oxysporum*, higher than that of the commercial agricultural fungicide hymexazol, whose inhibition rate was 56.12%.

## 1. Introduction

**Figure 1:**
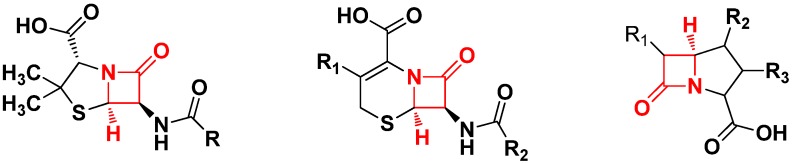
Penicillins, cephalosporins and carbapenems.

Chiral β-amino acid ester derivatives exhibit diverse biological properties such as antitumor, immunostimulating, and antiphlogistic activities [1,2,3,4]. They have been widely used as peptidomimetics and are found in many natural products. β-Lactams, which exhibit a wide range of biological properties such as antibiotic, antiviral, and protease inhibitor activities. Penicillin (Figure 1), the first antibiotic, has saved more than 50 million people’s life [5,6,7,8,9]. Cephalosporins and carbapenems (Figure 1) exhibit a broad spectrum of antibacterial activity. β-Amino acid ester derivatives can be used as building blocks for the synthesis of these types of antibiotics, making them useful in drug synthesis and other fields [10,11,12,13].

**Figure 2:**
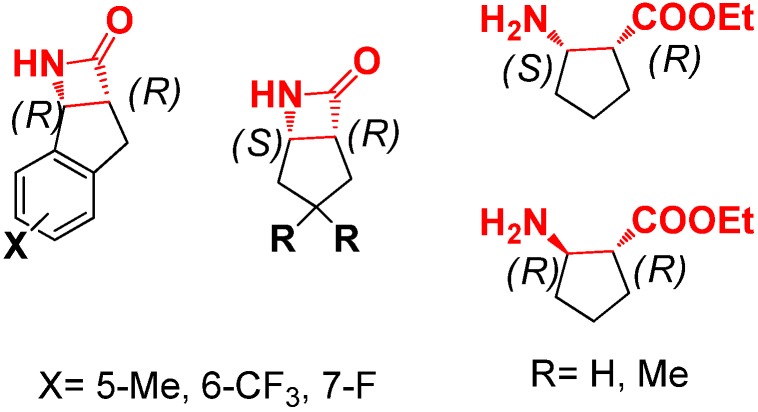
β-lactams and β-amino esters.

The asymmetric synthesis of β-amino ester derivatives or β-lactams has received much attention in organic synthesis over the past few years [3,14]. In 2011, Bull and coworkers reported the first example of an intramolecular cyclization of the ester enolate imine for the preparation of monocyclic β-lactams (Figure 2) [15].

The asymmetric Mannich reaction is one of the most general methods for the preparation of β-amino acid ester derivatives [16,17,18,19,20,21,22]. However, only simple aldehydes and amines have been used as Mannich reaction substrates. Numerous methods have been developed for improving its application [23,24,25,26,27,28,29,30,31,32,33,34,35,36,37], but imine substrates prepared from heteroaryl aldehydes were rarely reported.

Benzothiazoles have varied biological activities [38,39,40]. They are widely found in bioorganic and medicinal chemistry with applications in drug discovery and are still of great scientific interest nowadays [41]. Benzothiazole moieties are part of compounds showing numerous biological activities such as antibacterial, antimicrobial, anthelmintic, antitumor, anti-inflammatory properties [42,43].

Recently, we independently reported a chiral cinchona alkaloid-derived thiourea catalyst for enantioselective synthesis of chiral β-amino esters by Mannich-type reaction [44,45,46,47]. Herein, we report an extension of our previous study by using potentially bioactive benzothiophene and benzothiazole moieties [48,49,50] as the building blocks for the synthesis of chiral β-amino acid ester derivatives. The structures of these newly synthesized compounds were confirmed by ^1^H-NMR, ^13^C-NMR, IR spectra, and MS (HREI) analysis. The desired products **5a**–**5p** were obtained in good yields and high enantioselectivities (~86% yield, >99% ee) according to the optimized reaction conditions. Bioassays identified these newly compounds possessing weak to good antifungal activity. The inhibition rate of **5dr** (we used the postfixes “r” and “c” to distinguish between racemic and chiral compounds) against *F*. *oxysporum* was 60.53%, higher than the commercial agricultural fungicide *hymexazol* whose inhibition rate was 56.12%. Further experimental and mechanism of antifungal activity are underway.

## 2. Results and Discussion

### 2.1. Optimization of Reaction Conditions

**Scheme 1:**
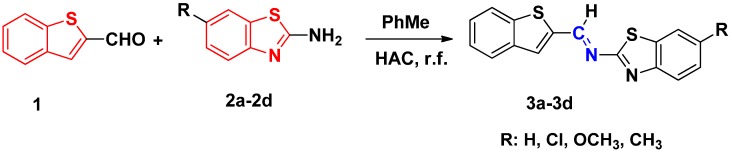
Synthesis of imines **3a**–**d**.

The general synthetic strategy for the preparation of imines by reaction of aldehydes with amino benzothiophene derivatives is outlined in Scheme 1. All products **3a**–**d** were characterized by spectroscopic methods.

**Figure 3:**
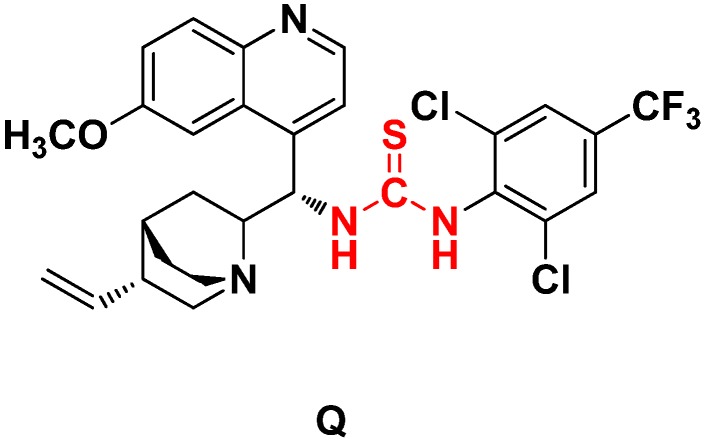
Structure of the cinchona alkaloid-derived thiourea catalyst.

Then, synthetically designed cinchona alkaloid thiourea **Q** (Figure 3) was used as the catalyst for asymmetric catalytic Mannich-type reaction of the imines and malonate ester. Catalyst **Q** bearing strong electron-withdrawing trifluoromethyl substituent on the benzene ring exhibited excellent catalytic activity because of its ability to promote the reaction through intermolecular hydrogen bond activation of the substrates. The effect of the reaction temperature, solvent, and catalyst loading was evaluated using catalyst **Q** (Table 1). Temperature had a pronounced effect on yield and enantioselectivity of the reactions. Use of other solvents, such as THF, acetone, and toluene, resulted in lower enatioselectivities compared to the use of dichloromethane (DCM) (Table 1, entries 1–5). The best result was achieved at room temperature using 10 mol% of catalyst **Q** in DCM.

**Table 1:** Optimization of reaction conditions using catalyst **Q**. 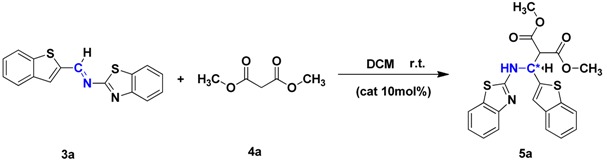

Entry	Temperature (°C)	Solvent	Catalyst (mol%)	Time (h)	Yield ^a^ (%)	ee ^b^ (%)
1	r.t.	THF	10	72	34	30
2	Reflux	THF	10	2	-	-
3	r.t.	PhMe	10	72	52	34
4	Reflux	PhMe	10	12	54	26
5	r.t.	Acetone	10	72	38	20
6	r.t.	DCM	10	72	61	78
7	35	DCM	10	24	67	56
8	r.t.	DCM	5	96	48	76
9	r.t.	DCM	20	72	72	78

^a^ Isolated yields after chromatographic purification; ^b^ Determined by HPLC analysis using Chiralpak IA.

Under the optimized reaction conditions, the synthetic scope of the reaction was investigated using different imines and malonate esters, and the results are listed in Table 2.

**Table 2:** Enantioselective Mannich-type reaction of imines and malonate esters. 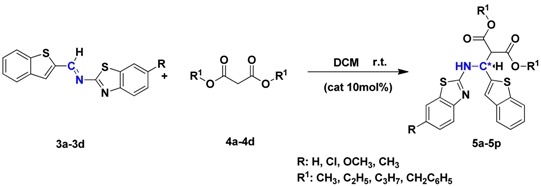

Entry	Products	R	R^1^	Time (h) ^a^	Yield (%) ^b^	ee (%) ^c^
1	**5ac**	6-H	−CH_3_	72	82	56
2	**5bc**	6-H	−C_2_H_5_	72	80	80
3	**5cc**	6-H	−C_3_H_7_	96	78	76
4	**5dc**	6-H	−CH_2_C_6_H_5_	96	76	92
5	**5ec**	6-Cl	−CH_3_	72	85	64
6	**5fc**	6-Cl	−C_2_H_5_	72	86	82
7	**5gc**	6-Cl	−C_3_H_7_	96	82	80
8	**5hc**	6-Cl	−CH_2_C_6_H_5_	96	78	>99
9	**5ic**	6-OCH_3_	−CH_3_	72	81	80
10	**5jc**	6-OCH_3_	−C_2_H_5_	72	80	80
11	**5kc**	6-OCH_3_	−C_3_H_7_	96	79	70
12	**5lc**	6-OCH_3_	−CH_2_C_6_H_5_	96	75	89
13	**5mc**	6-CH_3_	−CH_3_	72	81	78
14	**5nc**	6-CH_3_	−C_2_H_5_	72	76	76
15	**5oc**	6-CH_3_	−C_3_H_7_	96	76	80
16	**5pc**	6-CH_3_	−CH_2_C_6_H_5_	96	72	86

^a^ Reactions were performed using imine (0.50 mmol) and malonate ester (0.60 mmol) in DCM (3 mL) in the presence of 10 mol% catalyst **Q** at room temperature for 72–96 h; ^b^ Isolated yield after chromatographic purification; ^c^ Determined by HPLC analysis using Chiralpak IA.

The desired products **5a**–**p** were obtained in good yields and high enantioselectivities (~86% yield, >99% ee) using to the optimized reaction conditions. In addition, the enantioselectivity of **5dc**, **5hc**, **5lc** and **5pc** was higher than that of the other compounds, probably because of malonate ester was a benzyl ester, and steric hindrance affected the Mannich-type reaction between imines and benzyl esters.

### 2.2. Antifungal Activity

The antifungal activity of compounds **5** was assayed by the reported method [51,52]. As it can be seen from the results presented in Table 3, compound **5dr** possess medium antifungal activity against *G*. *zeae*, *C*. *mandshurica* and *F*. *oxysporum*, with inhibition rates of 40.67%, 41.44% and 60.53%, respectively.

**Table 3:** Fungicidal activity of the compounds **5ar**–**pr** at a concentration of 50 μg/mL.

Entry	Compound	Inhibition rate ^a^ *(%)*					
		*G*. *zeae*	*C*. *mandshurica*	*F*. *oxysporum*	*P*. *sasakii*	*P*. *infestans*	*S*. *sclerotiorum*
1	**5dr**	16.00 ± 0.1	9.76 ± 0.5	47.04 ± 1.1	38.16 ± 1.84	13.06 ± 1.5	37.42 ± 0.62
2	**5br**	11.67 ± 0.4	10.18 ± 0.7	37.17 ± 1.1	30.04 ± 2.31	9.70 ± 0.8	21.29 ± 0.76
3	**5cr**	6.03 ± 0.7	10.18 ± 0.8	19.74 ± 1.5	18.37 ± 1.18	4.1 ± 0.7	3.33 ± 0.5
4	**5dr**	40.67 ± 0.9	41.44 ± 0.7	60.53 ± 2.2	10.11 ± 1.22	1.12 ± 0.7	6.67 ± 0.6
5	**5er**	3.33 ± 0.5	11.66 ± 0.5	25.99 ± 1.1	16.01 ± 2.31	2.33 ± 0.8	3.20 ± 0.4
6	**5fr**	8.67 ± 0.5	16.45 ± 0.7	30.59 ± 0.7	18.37 ± 1.23	2.33 ± 0.8	2.30 ± 1.3
7	**5gr**	4.20 ± 0.5	13.13 ± 1.1	16.78 ± 2.3	9.80 ± 0.69	1.03 ± 0.8	5.67 ± 0.8
8	**5hr**	6.30 ± 0.7	11.66 ± 1.6	13.82 ± 2.3	13.18 ± 1.26	3.33 ± 0.5	3.33 ± 0.5
9	**5ir**	9.67 ± 0.9	18.97 ± 1.0	42.76 ± 2.6	11.21 ± 2.10	6.67 ± 0.6	21.29 ± 0.76
10	**5jr**	11.67 ± 0.6	12.71 ± 1.0	16.78 ± 1.7	13.07 ± 0.84	3.20 ± 0.4	3.33 ± 0.5
11	**5kr**	4.67 ± 0.7	11.23 ± 1.2	16.45 ± 1.5	15.90 ± 1.24	2.30 ± 1.3	6.67 ± 0.6
12	**5lr**	0.67 ± 0.6	17.18 ± 1.1	10.53 ± 2.1	3.33 ± 0.5	5.67 ± 0.8	3.20 ± 0.4
13	**5mr**	1.02 ± 0.7	10.87 ± 0.8	10.86 ± 1.2	6.67 ± 0.6	3.33 ± 0.5	2.30 ± 1.3
14	**5nr**	4.33 ± 0.8	10.50 ± 0.8	14.14 ± 0.5	3.20 ± 0.4	6.67 ± 0.6	5.67 ± 0.8
15	**5or**	4.33 ± 0.8	19.36 ± 1.5	14.14 ± 1.9	2.30 ± 1.3	3.20 ± 0.4	3.33 ± 0.5
16	**5pr**	3.33 ± 0.8	19.39 ± 0.3	29.93 ± 1.0	5.67 ± 0.8	2.30 ± 1.3	6.67 ± 0.6
17	**Hymexazole ^b^**	55.54 ± 3.9	49.61 ± 7.8	56.12 ± 4.1	51.21 ± 5.9	68.22 ± 2.4	77.51 ± 3.9
18	**DMSO**	0	0	0	0	0	0

^a^ Average of five replicates; ^b^ The commercial agricultural fungicide hymexazole was used for comparison of antifungal activity.

## 3. Experimental

### 3.1. Instruments and Chemicals

Melting points were determined using a XT-4 binocular microscope (Beijing Tech Instrument Co., Beijing, China) and were not corrected. IR spectra were recorded using a Bruker VECTOR 22 spectrometer in KBr disks. ^1^H- and ^13^C-NMR spectra were recorded using a JEOL-ECX 500 MHz NMR spectrometer at room temperature in CDCl_3_ or DMSO-d_6_ solvent using tetrametylsilane as the internal standard. Elemental analyses were performed using an Elementar Vario-III CHN analyzer. MS spectra were recorded using a VG Autospec-3000 spectrometer. Analytical TLC was performed using silica gel GF254 plates. Column chromatographic purifications were carried out using column chromatographic silica gel. All the reagents were purchased from commercial sources and used as received, unless otherwise noted. Reactions were performed under a positive dry argon pressure in oven-dried or flame-dried glassware equipped with a magnetic stir bar. Standard inert atmosphere techniques were used in handling all air and moisture sensitive reagents. All the reagents were of analytical reagent grade or in chemically pure form. All the solvents were dried, deoxygenated, and distilled prior to use.

### 3.2. Synthesis

#### 3.2.1. General Methods for Preparation of **3a**–**d**

6-Substituted-2-aminobenzothiazole (10 mmol) was dissolved in toluene (10 mL), and the resulting solution was magnetically stirred, followed by dropwise addition of benzothiophene-2-methanal (10 mmol) dissolved in toluene (10 mL) at room temperature. The resulting reaction mixture was refluxed after adding acetic acid (1.0 mL), and complete consumption of the starting materials was observed after 24 h. After the completion of the reaction, the solvent was removed by distillation under reduced pressure. The resulting residue was recrystallized using ethyl alcohol to afford products **3a**–**3d**.

#### 3.2.2. Characterization of **3a**–**d**

*N-(Benzo[b]thiophen-2-ylmethylene)benzo[d]thiazol-2-amine* (**3a**). Yellow solid; mp: 216−218 °C; yield: 78%; ^1^H-NMR (DMSO-*d*_6_) δ (ppm): 9.50–9.49 (m, 1H, 11-CH), 8.37-8.36 (m, 1H, 9-CH), 8.05–8.00 (m, 2H, 6-CH, 17-CH), 7.92–7.90 (m, 2H, 7-CH, 8-CH), 7.51–7.47 (m, 2H, 20-CH, 14-CH), 7.46–7.40 (m, 2H, 18-CH, 19-CH); ^13^C-NMR (DMSO-*d*_6_) δ (ppm): 171.1 (2-C), 161.5 (16-C), 151.8 (4-C), 142.2 (11-C), 141.2 (12-C), 139.6 (5-C), 136.5 (15-C), 134.8 (8-C), 128.6 (7-C), 127.3 (19-C), 126.5 (18-C), 125.9 (6-C), 125.5 (9-C), 123.7 (17-C), 123.2 (20-C), 122.3 (14-C); IR (KBr, cm^−1^) ν: 3051, 3022, 1587, 1558, 1456, 1417, 1149, 1120, 820, 723; MS (ESI): *m/z* = 295 [M+H]^+^, 317 [M+Na]^+^.

*N-(Benzo[b]thiophen-2-ylmethylene)-6-chlorobenzo[d]thiazol-2-amine* (**3b**). Yellow solid; mp: 229−231 °C; yield: 82%; ^1^H-NMR (DMSO-*d*_6_) δ (ppm): 9.47 (s, 1H, 6-CH), 8.37 (s, 1H, 11-CH), 8.23 (d, 1H, *J* = 5 Hz, 17-CH), 8.07–8.02 (m, 2H, 9-CH, 8-CH), 7.91(d, 1H, *J* = 10 Hz, 20-CH), 7.54–7.51 (m, 2H, 18-CH, 19-CH), 7.47–7.44 (m, 1H, 14-CH); ^13^C-NMR (DMSO-*d*_6_) δ (ppm): 162.1 (2-C), 150.6 (16-C), 142.1 (4-C), 141.1 (11-C), 139.6 (6-C), 136.8 (12-C), 136.2 (5-C), 130.2 (15-C), 128.7 (8-C), 127.8 (7-C), 126.5 (19-C), 126.0 (18-C), 125.4 (9-C), 124.4 (17-C), 123.8 (20-C), 122,7 (14-C); IR (KBr, cm^−1^) ν: 3053, 2843, 1600, 1564, 1479, 1174, 1143, 1126, 810, 748; MS (ESI): *m/z* = 329 [M+H]^+^, 351 [M+Na]^+^.

*N-(Benzo[b]thiophen-2-ylmethylene)-6-methoxybenzo[d]thiazol-2-amine* (**3c**). Yellow solid; mp: 181–183 °C; yield: 78%; ^1^H-NMR (DMSO-*d*_6_) δ (ppm): 9.44–9.43 (s, 1H, 11-CH), 8.35 (s, 1H, 17-CH), 8.09 (d, 1H, *J* = 5 Hz, 6-CH), 8.04 (d, 1H, *J* = 10 Hz, 8-CH), 7.85(d, 1H, *J* = 20 Hz, 9-CH), 7.68 (s, 1H, 20-CH), 7.56(t, 1H, *J* = 30 Hz, 19-CH), 7.49 (t, 1H, *J* = 30 Hz, 14-CH), 7.14–7.12 (m, 1H, 18-CH), 3.85 (s, 3H, 22-C-OCH_3_); ^13^C-NMR (DMSO-*d*_6_) δ (ppm): 168.5 (2-C), 160.3 (16-C), 158.0 (4-C), 146.1 (11-C), 141.8 (6-C), 141.4 (12-C), 139.6 (5-C), 136.2 (15-C), 135.8 (8-C), 128.4 (7-C), 126.3 (19-C), 125.9 (18-C), 123.9 (9-C), 123.7 (17-C), 116.6 (20-C), 105.6 (14-C), 56.3 (22-C); IR (KBr, cm^−1^) ν: 3055, 2843, 1598, 1570, 1477, 1458, 1425, 1265, 1224, 1126, 1118, 1053, 1018, 833, 746; MS (ESI): *m/z* = 325 [M+H]^+^, 347 [M+Na]^+^.

*N-(Benzo[b]thiophen-2-ylmethylene)-6-methylbenzo[d]thiazol-2-amine* (**3d**). Yellow solid; mp: 221–222 °C; yield: 78%; ^1^H-NMR (DMSO-*d*_6_) δ (ppm): 9.44 (d, 1H, *J* = 10 Hz, 11-CH), 8.33 (d, 1H, *J* = 10 Hz, 6-CH), 8.06–8.01 (m, 2H, 9-CH, 17-CH), 7.84–7.78 (m, 2H, 18-CH, 19-CH), 7.47–7.44 (m, 2H, 14-CH, 20-CH), 7.32(d, 1H, *J* = 10Hz, 8-CH), 2.46 (s, 3H, 21-CH_3_); ^13^C-NMR (DMSO-*d*_6_) δ (ppm): 170.0 (2-C), 160.9 (16-C), 149.8 (4-C), 141.9 (11-C), 141.3 (6-C), 139.6 (12-C), 136.2 (5-C), 135.8 (15-C), 134.8 (8-C), 128.7 (7-C), 128.5 (19-C), 126.4 (18-C), 125.9 (9-C), 123.7 (17-C), 122.8 (20-C), 122.5 (14-C), 21.7 (22-C); IR (KBr, cm^−1^) ν: 3045, 2845, 1573, 1539, 1471, 1427, 1346, 1118, 819, 744; MS (ESI): *m/z* = 309 [M+H]^+^, 331 [M+Na]^+^.

#### 3.2.3. General Method for the Preparation of **5a**–**p**

To a magnetically stirred solution of imine (0.50 mmol) in DCM (3 mL) in the presence of 10% catalyst **Q**, the malonate ester (0.7 mmol) was added dropwise at room temperature. The complete consumption of starting materials was observed after 72–96 h. After removing the solvent by reduced pressure distillation, the reaction mixture was subjected to column chromatography on silica gel (EA/PE = 1:7) to afford compounds **5a**–**p**.

#### 3.2.4. Characterization of **5a**–**p**

*Dimethyl 2-(benzo[b]thiophen-2-yl(benzo[d]thiazol-2-ylamino)methyl)malonate* (**5ar**). White solid; mp: 123–125 °C; yield: 82%; ^1^H-NMR (CDCl_3_): δ (ppm) 7.74 (d, 1H, *J* = 10 Hz, 24-CH), 7.68 (d, 1H, *J* = 10 Hz, 27-CH), 7.57 (t, 2H, *J* = 15 Hz, 14-CH, 17-CH), 7.31–7.25 (m, 4H, 25-CH, 26-CH, 15-CH, 16-CH), 7.10 (t, 1H, *J* = 15 Hz, 11-CH), 7.00 (s, 1H, NH), 6.14 (d, 1H, *J* = 5 Hz, 8-CH), 4.21 (d, 1H, *J* = 5 Hz, 2-CH), 3.73 (s, 6H, 28-CH_3_, 29-CH_3_); ^13^C-NMR (CDCl_3_): δ (ppm) 168.5 (20-C), 166.9 (1-C), 165.7 (3-C), 152.1 (10-C), 143.1 (13-C), 139.5 (22-C), 139.4 (23-C), 131.0 (12-C), 126.0 (15-C), 124.7 (16-C), 124.6 (25-C), 123.8 (26-C), 122.4 (24-C), 122.2 (27-C), 122.1 (14-C), 120.9 (17-C), 119.7 (11-C), 56.4 (8-C), 54.7 (2-C), 53.4 (28-C), 53.2 (29-C); MS (ESI): *m/z* = 427 [M+H]^+^, 449 [M+Na]^+^; MS (HREI): C_21_H_18_N_2_O_4_S_2_ Na for +, calculated 426.0708, found 426.0708; IR (KBr, cm^−1^) ν 3367, 2954, 1747, 1743, 1533, 1361, 1157, 1039, 842, 763, 723.

*(+) Dimethyl 2-(benzo[b]thiophen-2-yl(benzo[d]thiazol-2-ylamino)methyl)malonate* (**5ac**). This product was obtained as a white solid from a reaction catalyzed by **Q** (10 mol%) at 25 °C for 72 h; mp: 123–125 °C; yield: 81% after preparative column chromatography purification (silica gel, PE/ethyl ether = 7/1); 56.0% ee as determined by HPLC [Daicel Chiralpak IA, hexane/EtOH = 75/25, 1.0 mL/min, λ = 254 nm, tr (major) = 12.74 min, tr (minor) = 11.66 min]; 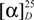 = +75.36 (c = 0.069 g/100 mL, CHCl_3_).

*Diethyl 2-(benzo[b]thiophen-2-yl(benzo[d]thiazol-2-ylamino)methyl)malonate* (**5br**). White solid; mp: 93–95 °C; yield: 83%; ^1^H-NMR (CDCl_3_)δ (ppm): 7.74 (d, 1H, *J* = 10 Hz, 24-CH), 7.68 (d, 1H, *J* = 10 Hz, 27-CH), 7.57 (t, 2H, *J* = 15Hz, 14-CH, 17-CH), 7.29-7.27 (m, 4H, 25-CH, 26-CH, 15-CH, 16-CH), 7.09 (t, 1H, *J* = 15 Hz, 11-CH), 7.04 (s, 1H, NH), 6.15 (d, 1H, *J* = 5Hz, 8-CH), 4.24 (d, 1H, *J* = 5Hz, 2-CH), 4.19–4.15 (m, 4H, 28-CH_2_, 29-CH_2_), 1.22–1.16 (m, 6H, 30-CH_3_, 31-CH_3_); ^13^C-NMR (CDCl_3_) δ (ppm): 168.1 (20-C), 166.6 (1-C), 165.8 (3-C), 152.2 (100-C), 143.3 (13-C), 139.5 (22-C), 131.0 (23-C), 126.0 (12-C), 124.8 (15-C), 124.8 (16-C), 124.6 (25-C), 123.7 (26-C), 122.4 (24-C), 122.1 (27-C), 122.0 (14-C), 120.9 (17-C), 119.6 (11-C), 62.5 (28-C), 62.2 (30-C), 56.7 (8-C), 54.7 (2-C), 14.0 (29-C), 13.8 (31-C); MS (ESI): *m/z* = 455 [M+H]^+^, 477 [M+Na]^+^; MS (HREI): C_23_H_22_N_2_O_4_S_2_ Na for +, calculated 454.1021, found 454.1017; IR (KBr, cm^−1^) ν 3371, 2978, 1735, 1720, 1592, 1481, 1373, 1278, 1199, 829, 756.

*(+) Diethyl 2-(benzo[b]thiophen-2-yl(benzo[d]thiazol-2-ylamino)methyl)malonate* (**5bc**). This product was obtained as a white solid from a reaction catalyzed by **Q** (10 mol%) at 25 °C for 72 h; mp: 93–95 °C; yield: 52% after preparative column chromatography purification (silica gel: PE/ethyl ether = 7/1); 80.0% ee as determined by HPLC [Daicel Chiralpak IA, hexane/EtOH = 75/25, 1.0 mL/min, λ = 254 nm, tr (major) = 8.65 min, tr (minor) = 10.57 min]; 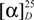 = +123.44 (c = 0.064 g/100 mL, CHCl_3_).

*Dipropyl 2-(benzo[b]thiophen-2-yl(benzo[d]thiazol-2-ylamino)methyl)malonate* (**5cr**). White solid; mp: 74–76 °C; yield: 68%; ^1^H-NMR (CDCl_3_) δ (ppm): 7.74 (d, 1H, *J* = 10 Hz, 24-CH), 7.67 (d, 1H, *J* = 10 Hz, 27-CH), 7.57 (t, 2H, *J* = 15 Hz, 14-CH, 17-CH), 7.31–7.26 (m, 4H, 25-CH, 26-CH, 15-CH, 16-CH), 7.09 (t, 1H, *J* = 15Hz, 11-CH), 7.02 (s, 1H, NH), 6.15 (d, 1H, *J* = 5 Hz, 8-CH), 4.20 (d, 1H, *J* = 5 Hz, 2-CH), 4.15–4.06 (m, 4H, 28-CH_2_, 29-CH_2_), 1.62–1.59 (m, 4H, 30-CH_2_, 31-CH_2_), 0.87–0.83 (m, 6H, 32-CH_3_, 33-CH_3_); ^13^C-NMR (CDCl_3_): δ (ppm) 168.3 (20-C), 166.7 (1-C), 165.7 (3-C), 152.2 (10-C), 143.4 (13-C), 139.5 (22-C), 131.0 (23-C), 126.0 (12-C), 124.6 (15-C), 124.6 (16-C), 124.5 (25-C), 123.7 (26-C), 122.3 (24-C), 122.1 (27-C), 122.0 (14-C), 120.9 (17-C), 119.7 (11-C), 68.0 (28-C), 67.8 (29-C), 56.6 (8-C), 54.7 (2-C), 21.9 (30-C), 21.8 (31-C), 10.3 (32-C), 10.2 (33-C); MS (ESI): *m/z* = 483 [M+H]^+^, 505 [M+Na]^+^; MS (HREI): C_25_H_26_N_2_O_4_S_2_ Na for +, calculated 482.1334, found 482.1337; IR (KBr, cm^−1^) ν 3348, 2964, 1743, 1718, 1533, 1481, 1392, 1286, 1199, 837, 756.

*(+) Dipropyl 2-(benzo[b]thiophen-2-yl(benzo[d]thiazol-2-ylamino)methyl)malonate* (**5cc**). This product was obtained as a white solid from a reaction catalyzed by **Q** (10 mol%) at 25 °C for 96 h; mp: 74–76 °C; yield: 76% after column chromatography purification (silica gel, PE/ethyl ether = 7/1); 76.0% ee as determined by HPLC [Daicel Chiralpak IA, hexane/EtOH = 75/25, 1.0 mL/min, λ = 254 nm, tr (major) = 7.45 min, tr (minor) = 9.54 min]; 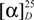 = +110.94 (c = 0.064 g/100 mL, CHCl_3_).

*Dibenzyl 2-(benzo[b]thiophen-2-yl(benzo[d]thiazol-2-ylamino)methyl)malonate* (**5dr**). White solid; mp: 117–119 °C; yield: 78%; ^1^H-NMR (CDCl_3_) δ (ppm): 7.72 (d, 1H, *J* = 5 Hz, 24-CH), 7.62 (d, 1H, *J* = 5 Hz, 27-CH), 7.57–7.53 (m, 2H, 14-CH, 31-CH), 7.31–7.27 (m, 3H, 32-CH, 33-CH, 34-CH), 7.22–7.17 (m, 5H, 35-CH, 37-CH, 38-CH, 39-CH, 40-CH), 7.15–7.10 (m, 7H, 41-CH, 17-CH, 25-CH, 26-CH, 15-CH, 16-CH, 11-CH), 6.94 (s, 1H, NH), 6.22 (d, 1H, *J* = 5 Hz, 8-CH), 5.19-5.06 (m, 4H, 28-CH_2_, 29-CH_2_), 4.30 (d, 1H, *J* = 5 Hz, 2-CH); ^13^C-NMR (CDCl_3_) δ (ppm): 168.0 (20-C), 166.5 (1-C), 166.3 (3-C), 165.5 (10-C), 152.1 (13-C), 143.1 (22-C), 139.5 (30-C), 139.4 (36-C), 134.7 (23-C), 134.6 (32-C), 131.1 (34-C), 128.7 (38-C), 128.6 (40-C), 128.5 (12-C), 128.5 (31-C), 128.4 (35-C), 128.3 (37-C), 128.2 (41-C), 128.1 (39-C), 126.0 (33-C), 124.6 (15-C), 124.5 (26-C), 123.8 (25-C), 122.4 (16-C), 122.2 (24-C), 122.0 (17-C), 120.9 (27-C), 119.7 (14-C), 68.2 (11-C), 67.9 (28-C), 56.7 (29-C), 54.5 (8-C), 42.3 (2-C); MS (ESI): *m/z* = 483 [M+H]^+^, 505 [M+Na]^+^; MS (HREI): C_33_H_26_N_2_O_4_S_2_ Na for +, calculated 578.1334, found 578.1348; IR (KBr, cm^−1^) ν 3338, 2953, 1739, 1716, 1593, 1454, 1381, 1199, 827, 750.

*(+) Dibenzyl 2-(benzo[b]thiophen-2-yl(benzo[d]thiazol-2-ylamino)methyl)malonate* (**5dc**). This product was obtained as a white solid from a reaction catalyzed by **Q** (10 mol%) at 25 °C for 96 h; mp: 117–119 °C; yield: 76% after column chromatography purification (silica gel: PE/ethyl ether = 7/1); 92.0% ee as determined by HPLC [Daicel Chiralpak IA, hexane/EtOH = 75/25, 1.0 mL/min, λ = 254 nm, tr (major) = 12.78 min, tr (minor) = 16.59 min]; 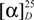 = +106.17 (c = 0.081 g/100 mL, CHCl_3_).

*Dimethyl 2-(benzo[b]thiophen-2-yl((6-chlorobenzo[d]thiazol-2-yl)amino)methyl) malonate* (**5er**). White solid; mp: 145–147 °C; yield: 82%; ^1^H-NMR (CDCl_3_) δ (ppm):7.74 (d, 1H, *J* = 10 Hz, 24-CH), 7.68 (d, 1H, *J* = 10Hz, 27-CH), 7.52 (s, 1H, 14-CH), 7.45 (d, 1H, *J* = 5 Hz, 17-CH), 7.33–7.22 (m, 4H, 25-CH, 26-CH, 15-CH, 11-CH), 7.07 (s, 1H, NH), 6.12 (s, 1H, 8-CH), 4.20 (d, 1H, *J* = 5 Hz, 2-CH), 3.73 (m, 6H, 29-CH_3_, 30-CH_3_); ^13^C-NMR (CDCl_3_): δ (ppm) 168.5 (20-C), 166.9 (1-C), 165.9 (3-C), 150.8 (10-C), 142.8 (13-C), 139.5 (22-C), 139.4 (23-C), 132.2 (12-C), 127.4 (15-C), 126.5 (16-C), 124.9 (25-C), 124.7 (26-C), 123.8 (24-C), 122.4 (27-C), 122.2 (14-C), 120.6 (17-C), 120.3 (11-C), 56.3 (8-C), 54.7 (2-C), 53.4 (29-C), 53.2 (30-C); MS (ESI): *m/z* = 461 [M+H]^+^, 483 [M+Na]^+^; MS (HREI): C_21_H_17_ClN_2_O_4_S_2_ Na for +, calculated 460.0318, found 460.0307; IR (KBr, cm^−1^) ν 3354, 2951, 1745, 1741, 1595, 1435, 1359, 1195, 835, 756.

*(+) Dimethyl 2-(benzo[b]thiophen-2-yl((6-chlorobenzo[d]thiazol-2-yl)amino)methyl) malonate* (**5ec**). This product was obtained as a white solid from a reaction catalyzed by **Q** (10 mol%) at 25 °C for 72 h; mp: 145–147 °C, yield: 82% after column chromatography purification (silica gel: PE/ethyl ether = 7/1); 64.0% ee as determined by HPLC [Daicel Chiralpak IA, hexane/EtOH = 75/25, 1.0 mL/min, λ = 254 nm, tr (major) = 13.95 min, tr (minor) = 20.91 min]; 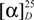 = +140.00 (c = 0.060 g/100 mL, CHCl_3_).

*Diethyl 2-(benzo[b]thiophen-2-yl((6-chlorobenzo[d]thiazol-2-yl)amino)methyl) malonate* (**5fr**). White solid; mp: 113–115 °C; yield: 78%; ^1^H-NMR (CDCl_3_) δ (ppm): 7.74 (d, 1H, *J* = 10 Hz, 24-CH), 7.68 (d, 1H, *J* = 10 Hz, 27-CH), 7.53–7.5 2(m, 1H, 14-CH), 7.45–7.42 (m, 1H, 17-CH), 7.33–7.22 (m, 4H, 26-CH, 15-CH, 16-CH, 11-CH), 7.07 (s, 1H, NH), 6.14 (s, 1H, 8-CH), 4.25 (d, 1H, *J* = 5 Hz, 2-CH), 4.22–4.14 (m, 4H, 28-CH_2_, 30-CH_2_), 1.22–1.16 (m, 6H, 29-CH_3_, 31-CH_3_); ^13^C-NMR (CDCl_3_) δ (ppm): 168.2 (20-C), 166.5 (1-C), 165.8 (3-C), 150.8 (10-C), 143.1(13-C), 139.4 (22-C), 132.2 (23-C), 127.3 (12-C), 126.5 (15-C), 124.8 (16-C), 124.7 (25-C), 124.6 (26-C), 123.8 (24-C), 122.4 (27-C), 122.0 (14-C), 120.6 (17-C), 120.3 (11-C), 62.6 (28-C), 62.3 (30-C), 56.5 (8-C), 54.7 (2-C), 14.0 (29-C), 13.9 (31-C); MS (ESI): *m/z* = 489 [M+H]^+^, 511 [M+Na]^+^; MS (HREI): C_23_H_21_ClN_2_O_4_S_2_ Na for +, calculated 488.0631, found 488.0625; IR (KBr, cm^−1^) ν 3354, 2978, 1743, 1720, 1593, 1444, 1093, 873, 761.

*(+) Diethyl 2-(benzo[b]thiophen-2-yl((6-chlorobenzo[d]thiazol-2-yl)amino)methyl) malonate* (**5fc**). This product was obtained as a white solid from a reaction catalyzed by **Q** (10 mol%) at 25 °C for 72 h; mp: 113–115 °C; yield: 76% after column chromatography purification (silica gel, PE/ethyl ether = 7/1); 82.0% ee as determined by HPLC [Daicel Chiralpak IA, hexane/EtOH = 75/25, 1.0 mL/min, λ = 254 nm, tr (major) = 11.14 min, tr (minor) = 19.58 min]; 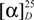 = +147.54 (c = 0.061 g/100 mL, CHCl_3_).

*Dipropyl 2-(benzo[b]thiophen-2-yl((6-chlorobenzo[d]thiazol-2-yl)amino)methyl) malonate* (**5gr**). White solid; mp: 92–94 °C; yield: 76%, ^1^H-NMR (CDCl_3_) δ (ppm): 7.75 (d, 1H, *J* = 5 Hz, 24-CH), 7.68 (d, 1H, *J* = 5 Hz, 27-CH), 7.44 (d, 1H, *J* = 10 Hz, 14-CH), 7.33–7.21 (m, 5H, 17-CH, 25-CH, 26-CH, 15-CH, 11-CH), 7.08 (s, 1H, NH), 6.14 (s, 1H, 8-CH), 4.18 (d, 1H, *J* = 5 Hz, 2-CH), 4.15–4.04 (m, 4H, 29-CH_2_, 30-CH_2_), 1.63–1.55 (m, 4H, 31-CH_2_, 32-CH_2_), 0.87–0.83 (m, 6H, 33-CH_3_, 34-CH_3_); ^13^C-NMR (CDCl_3_) δ (ppm): 168.3 (20-C), 166.6 (1-C), 165.8 (3-C), 150.8 (10-C), 143.1 (13-C), 139.5 (22-C), 139.4 (23-C), 132.2 (12-C), 127.3 (15-C), 126.4 (16-C), 124.6 (25-C), 124.5 (26-C), 123.7 (24-C), 122.4 (27-C), 122.0 (14-C), 120.5 (17-C), 120.3 (11-C), 68.1 (29-C), 67.8 (30-C), 56.5 (8-C), 54.6 (2-C), 21.9 (31-C), 21.8 (32-C), 10.3 (33-C), 10.2 (34-C); MS (ESI): *m/z* = 517 [M+H]^+^, 539 [M+Na]^+^; MS (HREI): C_25_H_25_ClN_2_O_4_S_2_ Na for +, calculated 516.0944, found 516.0932; IR (KBr, cm^−1^) ν 3354, 2964, 1747, 1722, 1593, 1479, 1199, 1170, 875, 754.

*(+) Dipropyl 2-(benzo[b]thiophen-2-yl((6-chlorobenzo[d]thiazol-2-yl)amino)methyl) malonate* (**5gc**). This product was obtained as a white solid from a reaction catalyzed by **Q** (10 mol%) at 25 °C for 96 h; mp: 92–94 °C, yield: 75% after column chromatography purification (silica gel, PE/ethyl ether = 7/1); 80.0% ee as determined by HPLC [Daicel Chiralpak IA, hexane/EtOH = 75/25, 1.0 mL/min, λ = 254 nm, tr (major) = 8.89 min, tr (minor) = 17.20 min]; 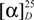 = +124.62 (c = 0.065 g/100 mL, CHCl_3_).

*Dibenzyl 2-(benzo[b]thiophen-2-yl((6-chlorobenzo[d]thiazol-2-yl)amino)methyl) malonate* (**5hr**). White solid; mp: 125–127 °C; yield: 82%, ^1^H-NMR (CDCl_3_) δ (ppm):7.72 (d, 1H, *J* = 5 Hz, 24-CH), 7.62 (d, 1H, *J* = 5 Hz, 27-CH), 7.51 (s, 1H, 14-CH), 7.41 (d, 1H, *J* = 10 Hz, 31-CH), 7.32–7.27 (m, 2H, 32-CH, 33-CH), 7.25–7.09 (m, 12H, 34-CH, 35-CH, 37-CH, 38-CH, 39-CH, 40-CH, 17-CH, 41-CH, 15-CH, 25-CH, 11-CH, 26-CH), 7.08 (s, 1H, NH), 6.19 (s, 1H, 8-CH), 5.20-5.04 (m, 4H, 28-CH_2_, 29-CH_2_), 4.28 ( d, 1H, *J* = 10 Hz, 2-CH); ^13^C-NMR (CDCl_3_) δ (ppm): 168.0 (20-C), 166.2 (1-C), 165.6 (3-C), 150.8 (10-C), 142.8 (13-C), 139.5 (22-C), 139.4 (30-C), 134.7 (36-C), 134.6 (23-C), 132.3 (32-C), 128.7 (34-C), 128.6 (38-C), 128.6 (40-C), 128.5 (12-C), 128.5 (31-C), 128.5 (35-C), 128.4 (37-C), 128.4 (41-C), 128.2 (39-C), 128.0 (33-C), 127.4 (15-C), 126.5 (26-C), 124.6 (25-C), 123.2 (16-C), 121.4 (24-C), 120.5 (17-C), 120.4 (27-C), 111.3 (14-C), 104.7 (11-C), 68.3 (28-C), 68.0 (29-C), 56.6 (8-C), 54.5 (2-C); MS (ESI): *m/z* = 613 [M+H]^+^, 635 [M+Na]^+^; MS (HREI): C_33_H_25_ClN_2_O_4_S_2_ Na for +, calculated 612.0944, found 612.0950; IR (KBr, cm^−1^) ν 3344, 2956, 1741, 1720, 1591, 1483, 1382, 1259, 1188, 827, 740.

*(+) Dibenzyl 2-(benzo[b]thiophen-2-yl((6-chlorobenzo[d]thiazol-2-yl)amino)methyl) malonate* (**5hc**). This product was obtained as a white solid from a reaction catalyzed by **Q** (10 mol%) at 25 °C for 96 h; mp: 125–127 °C; yield: 82% after column chromatography purification (Column chromatographic silica gel, PE/ethyl ether = 7/1); >99.0% ee as determined by HPLC [Daicel Chiralpak IA, hexane/EtOH =75/25, 1.0 mL/min, λ = 254 nm, tr (major) = 14.322 min, tr (minor) = 28.00 min]; 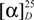 = +160.81 (c = 0.074 g/100 mL, CHCl_3_).

*Dimethyl 2-(benzo[b]thiophen-2-yl((6-methoxybenzo[d]thiazol-2-yl)amino) methyl)malonate* (**5ir**). White solid; mp: 78–80 °C; yield: 83%; ^1^H-NMR (CDCl_3_) δ (ppm):7.74–7.73 (d, 1H, *J* = 5 Hz, 24-CH), 7.68 (d, 1H, *J* = 5 Hz, 27-CH), 7.46 (d, 1H, *J* = 5 Hz, 14-CH), 7.32–7.25 (m, 3H, 17-CH, 25-CH, 26-CH), 7.10 (s, 1H, 15-CH), 6.88 (d, 1H, *J* = 5 Hz, 11-CH), 6.10 (s, 1H, NH), 5.29 (s, 1H, 8-CH), 4.20 (d, 1H, *J* = 5 Hz, 2-CH), 3.79 (s, 3H, 29-OCH_3_), 3.73 (s, 6H, 31-CH_3_, 31-CH_3_); ^13^C-NMR (CDCl_3_) δ (ppm): 168.4 (20-C), 167.0 (1-C), 164.0 (3-C), 155.6 (10-C), 146.3 (13-C), 143.3 (22-C), 139.5 (23-C), 139.4 (12-C), 132.0 (15-C), 124.6 (16-C), 124.5 (25-C), 123.8 (26-C), 122.4 (24-C), 122.0 (27-C), 120.0 (14-C), 113.7 (17-C), 105.3 (11-C), 56.4 (8-C), 56.0 (2-C), 54.7 (29-C), 53.0 (30-C), 53.1 (31-C); MS (ESI): *m/z* = 457 ([M+H]^+^), 479 ([M+Na]^+^); MS (HREI): C_22_H_20_N_2_O_5_S_2_ Na for +, calculated 456.0814, found 456.0847; IR (KBr, cm^−1^) ν 3346, 2951, 1743, 1722, 1541, 1473, 1355, 1277, 1058, 819, 742.

*(+) Dimethyl 2-(benzo[b]thiophen-2-yl((6-methoxybenzo[d]thiazol-2-yl)amino) methyl)malonate* (**5ic**). This product was obtained as a white solid from a reaction catalyzed by **Q** (10 mol%) at 25 °C for 72 h; mp: 78–80 °C; yield: 81% after column chromatography purification (silica gel, PE/ethyl ether = 7/1); 80.0% ee as determined by HPLC [Daicel Chiralpak IA, hexane/EtOH =75/25, 1.0 mL/min, λ = 254 nm, tr (major) = 15.67 min, tr (minor) = 18.49 min]; 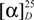 = +136.73 (c = 0.049 g/100 mL, CHCl_3_).

*Diethyl 2-(benzo[b]thiophen-2-yl((6-methoxybenzo[d]thiazol-2-yl)amino)methyl) malonate* (**5jr**). White solid; mp: 74–76 °C; yield: 81%; ^1^H-NMR (CDCl_3_) δ (ppm): 7.46 (t, 2H, *J* = 20 Hz, 24-CH, 27-CH), 7.41 (d, 1H, *J* = 5 Hz, 14-CH), 7.24 (t, 1H, *J* = 15 Hz, 17-CH), 7.18 (t, 1H, *J* = 15 Hz, 26-CH), 7.12 (d, 1H, *J* = 5 Hz, 15-CH), 6.72 (s, 1H, 11-CH), 6.67 (s, 1H, NH), 6.01 (s, 1H, 8-CH), 4.28 (d, 1H, *J* = 5 Hz, 2-CH), 4.24–4.14 (m, 4H, 28-CH_2_, 30-CH_2_), 3.80 (s, 3H, 25-OCH_3_), 1.20–1.17 (m, 6H, 29-CH_3_, 31-CH_3_); ^13^C-NMR (CDCl_3_) δ (ppm): 168.1 (20-C), 166.7 (1-C), 164.1 (3-C), 155.6 (10-C), 146.3 (13-C), 143.5 (22-C), 139.5 (23-C), 131.9 (12-C), 124.7 (15-C), 124.6 (16-C), 124.5 (25-C), 123.7 (26-C), 122.3 (24-C), 122.0 (27-C), 120.0 (14-C), 113.7 (17-C), 105.3 (11-C), 62.5 (28-C), 62.2 (30-C), 56.7 (8-C), 56.0 (2-C), 54.7 (25-C), 14.1 (29-C), 14.0 (31-C); MS (ESI): *m/z* = 485 [M+H]^+^, 507 [M+Na]^+^; MS HREI): C_24_H_24_N_2_O_5_S_2_ Na for +, calculated 484.1127, found 484.1118; IR (KBr, cm^−1^) ν 3321, 2956, 1747, 1718, 1543, 1458, 1276, 1193, 840, 750.

*(+) Diethyl 2-(benzo[b]thiophen-2-yl((6-methoxybenzo[d]thiazol-2-yl)amino)methyl) malonate* (**5jc**). This product was obtained as a white solid from a reaction catalyzed by **Q** (10 mol%) at 25 °C for 72 h; mp: 78–80 °C; yield: 81% after column chromatography purification (silica gel: PE/ethyl ether = 7/1); 80.0% ee as determined by HPLC [Daicel Chiralpak IA, hexane/EtOH = 75/25, 1.0 mL/min, λ = 254 nm, tr (major) = 11.75 min, tr (minor) = 16.49 min]; 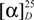 = +160.46 (c = 0.043 g/100 mL, CHCl_3_).

*Dipropyl 2-(benzo[b]thiophen-2-yl((6-methoxybenzo[d]thiazol-2-yl)amino)methyl) malonate* (**5kr**). White solid; mp: 99–101 °C; yield: 78%; ^1^H-NMR (CDCl_3_) δ (ppm): 7.74 (d, 1H, *J* = 10 Hz, 24-CH), 7.67 (d, 1H, *J* = 10 Hz, 27-CH), 7.45 (d, 1H, *J* = 10 Hz, 14-CH), 7.32–7.26 (m, 4H, 17-CH, 25-CH, 26-CH, 15-CH), 7.11 (d, 1H, *J* = 5 Hz, 11-CH), 6.89 (s, 1H, NH), 6.11 (d, 1H, *J* = 5 Hz, 8-CH), 4.18 (d, 1H, *J* = 5 Hz, 2-CH), 4.14–4.05 (m, 4H, 29-CH_2_, 30-CH_2_), 3.80 (s, 3H, 28-OCH_3_), 1.63–1.55 (m, 4H, 31-CH_2_, 32-CH_2_), 0.87–0.83 (m, 6H, 33-CH_3_, 34-CH_3_); ^13^C-NMR (CDCl_3_): δ (ppm) 168.3 (20-C), 166.7 (1-C), 164.0 (3-C), 155.5 (10-C), 146.3 (13-C), 143.6 (22-C), 139.5 (23-C), 132.0 (12-C), 124.9 (15-C), 124.8 (16-C), 124.5 (25-C), 123.7 (26-C), 122.3 (24-C), 122.0 (27-C), 120.0 (14-C), 113.6 (17-C), 105.3 (11-C), 68.0 (29-C), 67.7 (30-C), 56.7 (8-C), 56.0 (2-C), 54.6 (28-C), 21.9 (31-C), 21.8 (32-C), 10.3 (33-C), 10.2 (34-C); MS (ESI): *m/z* = 513 [M+H]^+^, 535 [M+Na]^+^; MS (HREI): C_26_H_28_N_2_O_5_S_2_ Na for +, calculated 512.1440, found 512.1429; IR (KBr, cm^−1^) ν 3331, 2962, 1747, 1718, 1543, 1276, 1190, 825, 752.

*(+) Dipropyl 2-(benzo[b]thiophen-2-yl((6-methoxybenzo[d]thiazol-2-yl)amino) methyl)malonate* (**5kc**). This product was obtained as a white solid from a reaction catalyzed by **Q** (10 mol%) at 25 °C for 96 h; mp: 99–101 °C; yield: 77% after column chromatography purification (silica gel, PE/ethyl ether = 7/1); 70.0% ee as determined by HPLC [Daicel Chiralpak IA, hexane/EtOH = 75/25, 1.0 mL/min, λ = 254 nm, tr (major) = 9.80 min, tr (minor) = 14.84 min]; 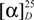 = +138.18 (c = 0.055 g/100 mL, CHCl_3_).

*Dibenzyl 2-(benzo[b]thiophen-2-yl((6-methoxybenzo[d]thiazol-2-yl)amino)methyl) malonate* (**5lr**). White solid; mp: 108–110 °C; yield: 78%; ^1^H-NMR (CDCl_3_) δ (ppm): 7.72 (d, 1H, *J* = 5 Hz, 24-CH), 7.62 (d, 1H, *J* = 5 Hz, 27-CH), 7.44 (d, 1H, *J* = 5 Hz, 14-CH), 7.31–7.28 (m, 3H, 31-CH, 32-CH, 33-CH), 7.25 (s, 1H, NH), 7.22–7.20 (m, 3H, 34-CH, 35-CH, 37-CH), 7.17–7.10 (m, 8H, 38-CH, 39-CH, 40-CH, 17-CH, 41-CH, 15-CH, 26-CH, 11-CH), 6.89 (d, 1H, *J* = 10 Hz, 26-CH), 6.18 (s, 1H, 8-CH), 5.16–5.06 (m, 4H, 28-CH_2_, 29-CH_2_), 4.29 (d, 1H, *J* = 5 Hz, 2-CH), 3.81 (s, 3H, 25-OCH_3_); ^13^C-NMR (CDCl_3_): δ (ppm) 167.9 (20-C), 166.3 (1-C), 163.8 (3-C), 155.6 (10-C), 146.4 (13-C), 143.2 (22-C), 139.5 (30-C), 134.8 (36-C), 134.7 (23-C), 132.1 (32-C), 128.7 (34-C), 128.6 (38-C), 128.5 (40-C), 128.5 (12-C), 128.5 (31-C), 128.4 (35-C), 128.4 (37-C), 128.3 (41-C), 128.2 (39-C), 128.1 (33-C), 124.4 (15-C), 123.1 (26-C), 121.3 (25-C), 120.1 (16-C), 113.7 (24-C), 111.3 (17-C), 105.3 (27-C), 104.7 (14-C), 68.2 (11-C), 67.9 (28-C), 67.8 (29-C), 56.7 (25-C), 56.0 (8-C), 54.5 (2-C); MS(ESI): *m/z* = 609 [M+H]^+^, 631 [M+Na]^+^; MS (HREI): C_34_H_28_N_2_O_5_S_2_ Na for +, calculated 608.1440, found 608.1440; IR (KBr, cm^−1^) ν 3361, 2358, 1747, 1716, 1541, 1471, 1222, 1101, 817, 740.

*(+)Dibenzyl 2-(benzo[b]thiophen-2-yl((6-methoxybenzo[d]thiazol-2-yl)amino) methyl)malonate* (**5lc**). This product was obtained as a white solid from a reaction catalyzed by **Q** (10 mol%) at 25 °C for 96 h; mp: 108–110 °C; yield: 78% after column chromatography purification (silica gel, PE/ethyl ether = 7/1); 89.0% ee as determined by HPLC [Daicel Chiralpak IA, hexane/EtOH =75/25, 1.0 mL/min, λ = 254 nm, tr (major) = 15.58 min, tr (minor) = 23.21 min]; 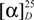 = +125 (c = 0.036 g/100 mL, CHCl_3_).

*Dimethyl 2-(benzo[b]thiophen-2-yl((6-methylbenzo[d]thiazol-2-yl)amino)methyl) malonate* (**5mr**). White solid; mp: 110–112 °C; yield: 82%; ^1^H-NMR (CDCl_3_) δ (ppm): 7.75 (d, 1H, *J* = 5 Hz, 24-CH), 7.69 (d, 1H, *J* = 5 Hz, 27-CH), 7.45 (d, 1H, *J* = 5 Hz, 14-CH), 7.38 (s, 1H, 17-CH), 7.33–7.27 (m, 3H, 25-CH, 26-CH, 15-CH), 7.09 (d, 1H, *J* = 10 Hz, 11-CH), 6.85 (s, 1H, NH), 6.12 (s, 1H, 8-CH), 4.21 (d, 1H, *J* = 5 Hz, 2-CH), 3.73 (s, 6H, 29-CH_3_, 30-CH_3_), 2.38 (s, 3H, 28-CH_3_); ^13^C-NMR (CDCl_3_) δ (ppm): 168.4 (20-C), 167.0 (1-C), 165.0 (3-C), 150.0 (10-C), 143.7 (13-C), 139.5 (22-C), 132.0 (23-C), 131.0 (12-C), 127.2 (15-C), 124.8 (16-C), 124.6 (25-C), 124.5 (26-C), 123.8 (24-C), 122.4 (27-C), 122.0 (14-C), 121.1 (17-C), 119.3 (11-C), 56.4 (8-C), 54.7 (2-C), 53.3 (29-C), 53.1 (30-C), 21.3 (28-C); MS (ESI): *m/z* = 441 [M+H]^+^, 463 [M+Na]^+^; MS (HREI): C_22_H_20_N_2_O_4_S_2_ Na for +, calculated 440.0864, found 440.0852; IR (KBr, cm^−1^) ν 3361, 2951, 1743, 1732, 1541, 1479, 1359, 1233, 1161, 815, 748.

*(+) Dimethyl 2-(benzo[b]thiophen-2-yl((6-methylbenzo[d]thiazol-2-yl)amino)methyl) malonate* (**5mc**). This product was obtained as a white solid from a reaction catalyzed by **Q** (10 mol%) at 25 °C for 72 h; mp: 110–112 °C; yield: 82% after column chromatography purification (silica gel: PE/ethyl ether = 7/1); 78.0% ee as determined by HPLC [Daicel Chiralpak AD-H, hexane/EtOH = 75/25, 1.0 mL/min, λ = 254 nm, tr (major) = 26.94 min, tr (minor) = 24.52 min]; 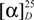 = +125.93 (c = 0.054 g/100 mL, CHCl_3_).

*Diethyl 2-(benzo[b]thiophen-2-yl((6-methylbenzo[d]thiazol-2-yl)amino)methyl) malonate* (**5nr**). White solid; mp: 115–117 °C; yield: 80%; ^1^H-NMR (CDCl_3_) δ (ppm): 7.78–7.74 (m, 1H, 24-CH), 7.76–7.67 (m, 1H, 27-CH), 7.47–7.43 (m, 1H, 14-CH), 7.40–7.38 (m, 1H, 17-CH), 7.32–7.26 (m, 3H, 26-CH, 15-CH, 16-CH), 7.12–7.08 (m, 1H, 11-CH), 6.92 (s, 1H, NH), 6.14 (s, 1H, 8-CH), 4.26–4.22 (m, 1H, 2-CH), 4.20–4.16 (m, 4H, 28-CH_2_, 30-CH_2_), 2.41–2.38 (m, 3H, 25-CH_3_), 1.24–1.17 (m, 6H, 29-CH_3_, 31-CH_3_); ^13^C-NMR (CDCl_3_) δ (ppm): 168.1 (20-C), 166.7 (1-C), 165.1 (3-C), 150.2 (10-C), 150.0 (13-C), 143.5 (22-C), 139.5 (23-C), 132.1 (12-C), 131.0 (15-C), 127.2 (16-C), 124.5 (25-C), 123.7 (26-C), 122.3 (24-C), 121.9 (27-C), 121.2 (14-C), 121.0 (17-C), 119.2 (11-C), 62.5 (28-C), 62.2 (30-C), 56.6 (8-C), 54.7 (2-C), 21.3 (25-C), 14.3 (29-C), 14.0 (31-C); MS (ESI): *m/z* = 469 [M+H]^+^, 491 [M+Na]^+^; MS (HREI): C_24_H_24_N_2_O_4_S_2_ Na for +, calculated 468.1177, found 468.1179; IR (KBr, cm^−1^) ν 3363, 2974, 1741, 1714, 1541, 1485, 1238, 1157, 1095, 817, 763.

*(+) Diethyl 2-(benzo[b]thiophen-2-yl((6-methylbenzo[d]thiazol-2-yl)amino)methyl) malonate* (**5nc**). this product was obtained as a white solid from a reaction catalyzed by **Q** (10 mol%) at 25 °C for 72 h; mp: 115–117 °C; yield: 80% after column chromatography purification (silica gel, PE/ethyl ether = 7/1); 76.0% ee as determined by HPLC [Daicel Chiralpak IA, Hexane/EtOH = 75/25, 1.0 mL/min, λ = 254 nm, tr (major) = 9.90 min, tr (minor) = 11.94 min]; 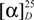 = +204.17 (c = 0.024 g/100 mL, CHCl_3_).

*Dipropyl 2-(benzo[b]thiophen-2-yl((6-methylbenzo[d]thiazol-2-yl)amino)methyl) malonate* (**5or**). White solid; mp: 109–111 °C; yield: 76%; ^1^H-NMR (CDCl_3_) δ (ppm):7.73 (d, 1H, *J* = 10 Hz, 24-CH), 7.66 (d, 1H, *J* = 10 Hz, 27-CH), 7.42 (t, 1H, *J* = 15 Hz, 14-CH), 7.37 (s, 1H, 17-CH), 7.27–7.25 (m, 3H, 25-CH, 26-CH, 15-CH), 7.09–7.06 (m, 1H, 11-CH), 6.91 (s, 1H, NH), 6.11 (s, 1H, 8-CH), 4.17 (d, 1H, *J* = 10 Hz, 2-CH), 4.12–4.06 (m, 4H, 29-CH_2_, 30-CH_2_), 2.37 (s, 3H, 28-CH_3_), 1.60–1.57 (m, 4H, 31-CH_2_, 32-CH_2_), 0.85–0.82 (m, 6H, 33-CH_3_, 34-CH_3_); ^13^C-NMR (CDCl_3_) δ (ppm): 168.3 (20-C), 166.7 (1-C), 165.0 (3-C), 150.0 (10-C), 143.5 (13-C), 139.5 (22-C), 131.9 (23-C), 131.0 (12-C), 127.1 (15-C), 124.7 (16-C), 124.6 (25-C), 124.5 (26-C), 123.7 (24-C), 122.3 (27-C), 121.9 (14-C), 120.9 (17-C), 119.2 (11-C), 68.0 (29-C), 67.7 (30-C), 56.6 (8-C), 54.7 (2-C), 21.9 (28-C), 21.8 (31-C), 21.3 (32-C), 10.2 (33-C), 10.0 (34-C); MS (ESI): *m/z* = 497 [M+H]^+^, 519 [M+Na]^+^; MS (HREI): C_26_H_28_N_2_O_4_S_2_ Na for +, calculated 496.1490, found 496.1491; IR (KBr, cm^−1^) ν 3332, 2962, 1740, 1724, 1541, 1238, 1157, 812, 763.

*(+) Dipropyl 2-(benzo[b]thiophen-2-yl((6-methylbenzo[d]thiazol-2-yl)amino)methyl) malonate* (**5oc**). This product was obtained as a white solid from a reaction catalyzed by **Q** (10 mol%) at 25 °C for 96 h; mp: 109–111 °C; yield: 75% after column chromatography purification (silica gel, PE/ethyl ether = 7/1); 80.0% ee as determined by HPLC [Daicel Chiralpak IA, Hexane/EtOH = 75/25, 1.0 mL/min, λ = 254 nm, tr (major) = 8.32 min, tr (minor) = 10.77 min]; 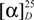 = +78.12 (c = 0.064 g/100 mL, CHCl_3_).

*Dibenzyl 2-(benzo[b]thiophen-2-yl((6-methylbenzo[d]thiazol-2-yl)amino)methyl) malonate* (**5pr**). White solid; mp: 126–128 °C; yield: 80%; ^1^H-NMR (CDCl_3_) δ (ppm): 7.71 (d, 1H, *J* = 5 Hz, 24-CH), 7.61 (d, 1H, *J* = 5 Hz, 27-CH), 7.43 (d, 1H, *J* = 5 Hz, 14-CH), 7.35 (s, 1H, NH), 7.30–7.27 (m, 2H, 31-CH, 32-CH), 7.19–7.08 (m, 13H, 33-CH, 34-CH, 35-CH, 37-CH, 38-CH, 39-CH, 40-CH, 17-CH, 41-CH, 15-CH, 16-CH, 11-CH, 26-CH), 6.19 (s, 1H, 8-CH), 5.16–5.05 (m, 4H, 28-CH_2_, 29-CH_2_), 4.29 (d, 1H, *J* = 5 Hz, 2-CH), 2.38 (s, 3H, 25-CH_3_); ^13^C-NMR (CDCl_3_) δ (ppm): 167.9 (20-C), 166.4 (1-C), 164.8 (3-C), 150.0 (10-C), 143.2 (13-C), 139.5 (22-C), 134.8 (30-C), 132.0 (36-C), 131.1 (23-C), 131.1 (32-C), 128.7 (34-C), 128.7 (38-C), 128.6 (40-C), 128.6 (12-C), 128.5 (31-C), 128.5 (35-C), 128.2 (37-C), 128.2 (41-C), 128.1 (39-C), 128.1 (33-C), 127.2 (15-C), 127.2 (26-C), 124.7 (25-C), 123.1 (16-C), 121.4 (24-C), 121.0 (17-C), 119.3 (27-C), 111.3 (14-C), 104.7 (11-C), 68.7 (28-C), 67.9 (29-C), 57.2 (8-C), 54.3 (2-C), 21.4 (25-C); MS (ESI): *m/z* = 593 [M+H]^+^, 615 [M+Na]^+^; MS (HREI): C_34_H_28_N_2_O_4_S_2_ Na for +, calculated 592.1490, found 592.1491; IR (KBr, cm^−1^) ν 3346, 2954, 1743, 1724, 1533, 1481, 1255, 1188, 812, 744.

*(+) Dibenzyl 2-(benzo[b]thiophen-2-yl((6-methylbenzo[d]thiazol-2-yl)amino)methyl) malonate* (**5pc**). This product was obtained as a white solid from a reaction catalyzed by **Q** (10 mol%) at 25 °C for 96 h; mp: 126–128 °C; yield: 79% after column chromatography purification (silica gel, PE/ethyl ether = 7/1); 86.0% ee as determined by HPLC [Daicel Chiralpak IA, hexane/EtOH = 75/25, 1.0 mL/min, λ = 254 nm, tr (major) = 12.63 min, tr (minor) = 17.04 min]; 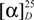 = +168.96 (c = 0.029 g/100 mL, CHCl_3_).

### 3.3. Antifungal Activity Section

The antifungal activity of all synthesized compounds was tested against six pathogenic fungi, *G*. *azeae*, *F*. *oxysporum*, *C*. *mandshurica*, *P*. *sasakii*, *P*. *infestans and S*. *sclerotiorum* through the poison plate technique. All the compounds were dissolved in dimethyl sulfoxide (DMSO, 10 mL) before mixing with potato dextrose agar (PDA, 90 mL). The compounds were tested at a concentration of 50 μg/mL. All fungal species were incubated in PDA at 27 ± 1 °C for 5 day to obtain new mycelium for antifungal assay. Mycelia dishes approximately 4 mm in diameter were cut from the culture medium. One of the specimens was picked up with a sterilized inoculation needle and then inoculated in the center of the PDA plate aseptically. The inoculated plates were incubated at 27 ± 1°C for 5 day. DMSO in sterile distilled water served as the control, whereas hymexazole acted as the positive control. Three replicates were conducted for each treatment. The radial growth of the fungal colonies was measured, and the data were statistically analyzed. The inhibitory effects of the test compounds *in vitro* against these fungi were calculated as follows:

*I*(%) = [(*C* − *T*)/(*C* - 0.4)] × 100

where C represents the diameter of fungal growth on untreated PDA, T represents the diameter of fungi on treated PDA, and I is the inhibitory rate.

## 4. Conclusions

Sixteen pairs of chiral β-amino acid ester derivatives containing benzothiophene and benzothiazole units were designed and synthesized. The desired products **5a**–**p** were obtained in good yields and high enantioselectivities (~86% yield, >99% ee) according to the optimized reaction conditions. The enantioselectivity of **5dc**, **5hc**, **5lc** and **5pc** was higher than the other compounds, probably because the malonate ester was a benzyl ester, and steric hindrance affected the Mannich-type reaction between the imines and benzyl esters. Bioassays identified these new compounds as possessing weak to good antifungal activity. The inhibition rate of **5dr** against *F*. *oxysporum* was 60.53%, higher than the commercial agricultural fungicide hymexazole whose inhibition rate was 56.12% at a concentration of 50 μg/mL. Further experimental studies of the mechanism of antifungal activity are underway.
